# Key Drivers Associated with Effective Sepsis Care on a Surgical Unit

**DOI:** 10.1097/pq9.0000000000000628

**Published:** 2023-01-16

**Authors:** Richele Koehler, Meghan Burke, Elise Rolison, Jennifer Newman, Justin M Lockwood

**Affiliations:** From the * Division of Pediatric Surgery, Department of Surgery, University of Colorado School of Medicine, Aurora, CO; † Surgical Specialties & Rehabilitation, Children’s Hospital Colorado, Aurora, CO; ‡ Clinical Effectiveness Team, Children’s Hospital Colorado, Aurora, CO; § Patient Safety Team, Children’s Hospital Colorado, Aurora, CO; ¶ Section of Hospital Medicine, Department of Pediatrics, University of Colorado School of Medicine, Aurora, CO.

## Abstract

In 2020, we observed an increase in inpatient postoperative patient deteriorations. Preliminary case reviews suggested gaps in sepsis care.

**Objectives:**

To identify key drivers associated with effective care escalation and sepsis care in the inpatient surgical setting as part of the initial planning phase of a large improvement initiative.

**Methods:**

Our multidisciplinary team included surgical nurses, surgical providers, medical providers, patient safety experts, subject matter experts in pediatric sepsis, and subject matter experts in escalation of care. This team formally reviewed cases involving postoperative deterioration to identify causes of gaps in sepsis care and applied its learnings to the development of a key driver diagram. Themes from the key driver diagram included (1) Enhance recognition of deterioration and subsequent escalation of care and (2) Optimize postoperative patient placement. The team hosted a mini-Kaizen event with frontline team members focused on the first theme, the output of which included consensus agreement on two focus areas (Escalation Chain of Command & Shared Situation Awareness) and a fishbone diagram to identify/prioritize opportunities in postoperative care escalation.

**Results:**

Causes of gaps in sepsis care included barriers to care escalation that could limit generalizability of sepsis resources to the inpatient surgical setting. Figure 1 shows the key driver diagram.

**Conclusion:**

We aim to highlight challenges associated with postoperative sepsis care in an inpatient pediatric surgical setting.

**Fig. 1.**
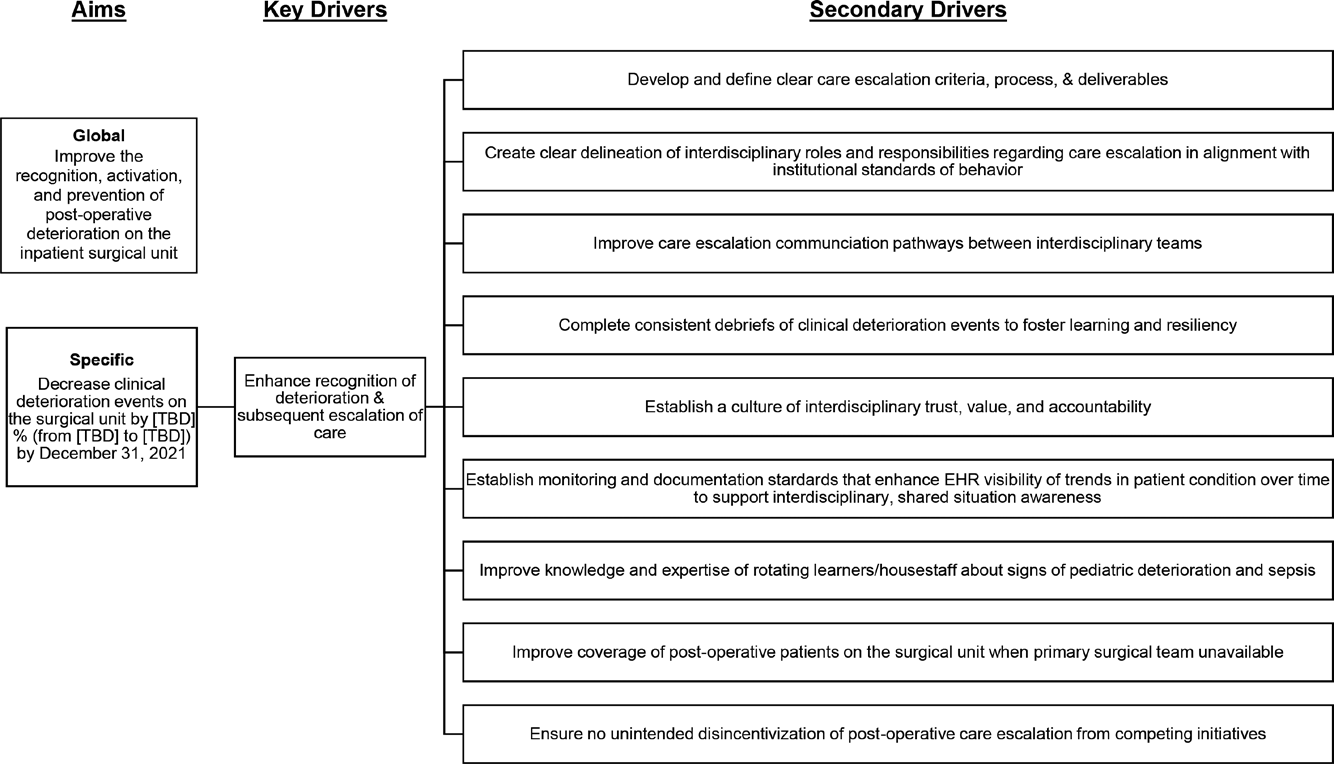
Key driver diagram for effective postoperative care escalation on the surgical unit (patient placement optimization key driver not shown).

